# In vivo reproducibility of tooth shade determination on intraoral scans compared to the dental spectrophotometric method

**DOI:** 10.1007/s00784-025-06240-5

**Published:** 2025-03-15

**Authors:** Louisa Kupke, Christian Schwahn, Karl-Friedrich Krey, Anja Ratzmann

**Affiliations:** 1 Department of Orthodontics, Dental School, University Medicine, Walther-Rathenau-Straße 42, 17475 Greifswald, Germany; 2 Department of Restorative Dentistry, Dental School, University Medicine, Walther-Rathenau- Straße 42, 17475 Greifswald, Germany

**Keywords:** Digital orthodontics, Digital diagnostics, Intraoral scanner, Digital tooth shade determination

## Abstract

**Objective:**

The purpose of the study was to assess the reproducibility of the color analysis determination module of an intraoral scanner in comparison with spectrophotometric tooth color measurement concerning ΔE and d(0M1).

**Materials and methods:**

The color of teeth (13–23) of 35 participants was electronically measured with the intraoral scanner CEREC Primescan AC compared to the spectrophotometer VITA Easyshade V with positioning splints. VITA classical A1-D4® served as a reference system.

**Results:**

Concerning repeated measurements, both methods achieved comparable intra-method (very good) and inter-method (good) reproducibility in terms of ΔE and d(0M1) for statistics of agreement and reliability. Intra-method there were differences in favor of the spectrophotometer. For ΔE < 3.7 and d(0M1) < 2.7/< 3.7, both methods recorded excellent agreement. The standard error of measurement of the intraoral scanner was larger and the smallest detectable color difference was smaller for the spectrophotometer. The Bland-Altman plots showed a similar distribution.

**Conclusion:**

The intra- and inter-method reproducibility of tooth color measurement with the intraoral scanner was comparable to the spectrophotometric method.

**Clinical relevance:**

Tooth shade determination with intraoral scanners can be regarded as an alternative to spectrophotometric methods in clinical practice.

**Trial registration:**

Deutsches Register klinischer Studien (DRKSID DRKS00025498, registration date 16.07.2021).

## Background

The clinical application of tooth shade assessment is important for esthetic, restorative, and preventive dentistry including the diagnosis and documentation of intrinsic and extrinsic tooth shade changes [1]. For example, fluorosis, enamel hypoplasia, amelogenesis- or dentinogenesis imperfecta, and external influences can be quantified and categorized using electronic tooth color measurement methods [2]. In orthodontics tooth color changes caused by bonding and debonding procedures can be investigated [3]. Thus color changes caused by fixed orthodontic appliances, which can be attributed to the interaction of bonding materials with food ingredients, UV-light, or corrosion products, can be examined [4].

The implementation of digital technologies in dentistry is fundamentally redesigning dental workflows. Intraoral scanner systems are used to generate digital data sets through three-dimensional intraoral images. The further development of hardware and software programs for intraoral scanning systems is enabling an increasing number of possible applications [5–7]. Since 2017, a color determination module was added to the software of some intraoral scanning systems which enables the direct assessment of tooth color [8]. Since then, intraoral scanners have been gaining importance as a color determination method next to visual tooth color determination and electronic color measuring devices such as dental spectrophotometers [7]. The most common method in clinical practice is still the visual tooth color assessment by comparing prefabricated color scales or using measuring devices such as colorimeters, spectrophotometers, or digital imaging systems with different software systems [7, 9, 10]. There is currently a small amount of research available concerning the reproducibility of tooth shade determination with intraoral scanning systems [6].

Tooth colors can be assessed using different color spaces. The Munsell color system, which defines color as a three-dimensional phenomenon, describes three dimensions: hue, value, and chroma [11]. According to this, a quantifiable description of tooth colors based on color coordinates within these three-dimensional color spaces is possible. 1976 the International Commission on Illumination published the CIE L*a*b* system which describes the lightness (L*), a point on the red-green axis (a*), and a point on the blue-yellow axis (b*) within a cartesian coordinate system. Analogous to the CIE L*a*b* system, the modified L*C*h color space is used, which specifies lightness (L*), chroma (C*), and hue (h*). Delta E, the Euclidean color distance, enables the quantification of color differences. Acknowledging that clinical settings necessitate higher ΔE values for the reliable detection of color variations compared to controlled experimental conditions we used perceptibility thresholds of ΔE < 2.7 and ΔE < 3.7. To overcome the limitations of ΔE, which is not applicable for examining disagreement patterns or estimating measurement variability through the intraclass correlation coefficient (ICC), we utilized the distance of each shade from 0M1 of the VITA 3D Master system, denoted as d(0M1) [10]. Specifically, d(0M1) can be applied to important graphical and statistical methods for assessing validity and reproducibility, which ΔE cannot. These methods include Bland-Altman plots to examine patterns of disagreement and ICC to estimate reliability. Because d(0M1) does not differentiate between shades equidistant from M1, it serves as a complementary rather than competing metric to ΔE [10]. It can therefore be stated that while we based our study on the clinically predominant VITA classical system, all color values were converted to system-independent CIE L*a*b* values, allowing us to apply the validated d(0M1) metric.

### Objective

This study aimed to investigate the reproducibility of the electronic tooth shade determination with the intraoral scanning system CEREC Primescan AC (Sirona Dental Systems GmbH, Bensheim, Germany) compared to the spectrophotometer VITA Easyshade^®^ V (VITA Zahnfabrik, Bad Säckingen, Germany) concerning ΔE < 2.7 / ΔE < 3.7 and d(0M1).

## Materials and methods

### Subjects and clinical procedure

Thirty-five participants (13 male, 22 female, average age 27) took part in this study. All of them gave informed consent. The inclusion criteria were a permanent dentition and good oral hygiene. Participants with any type of general diseases or dentures were excluded. For the tooth color measurements non-vital teeth, restoration materials, alterations of dental hard tissues, carious or periodontal disease, and previous bleaching procedures were also excluded. Approval of the Ethics Committee of the University Medical Center Greifswald was given (Reg. No.: BB 071/14).

### Electronic tooth color assessment

**Fig. 1:**
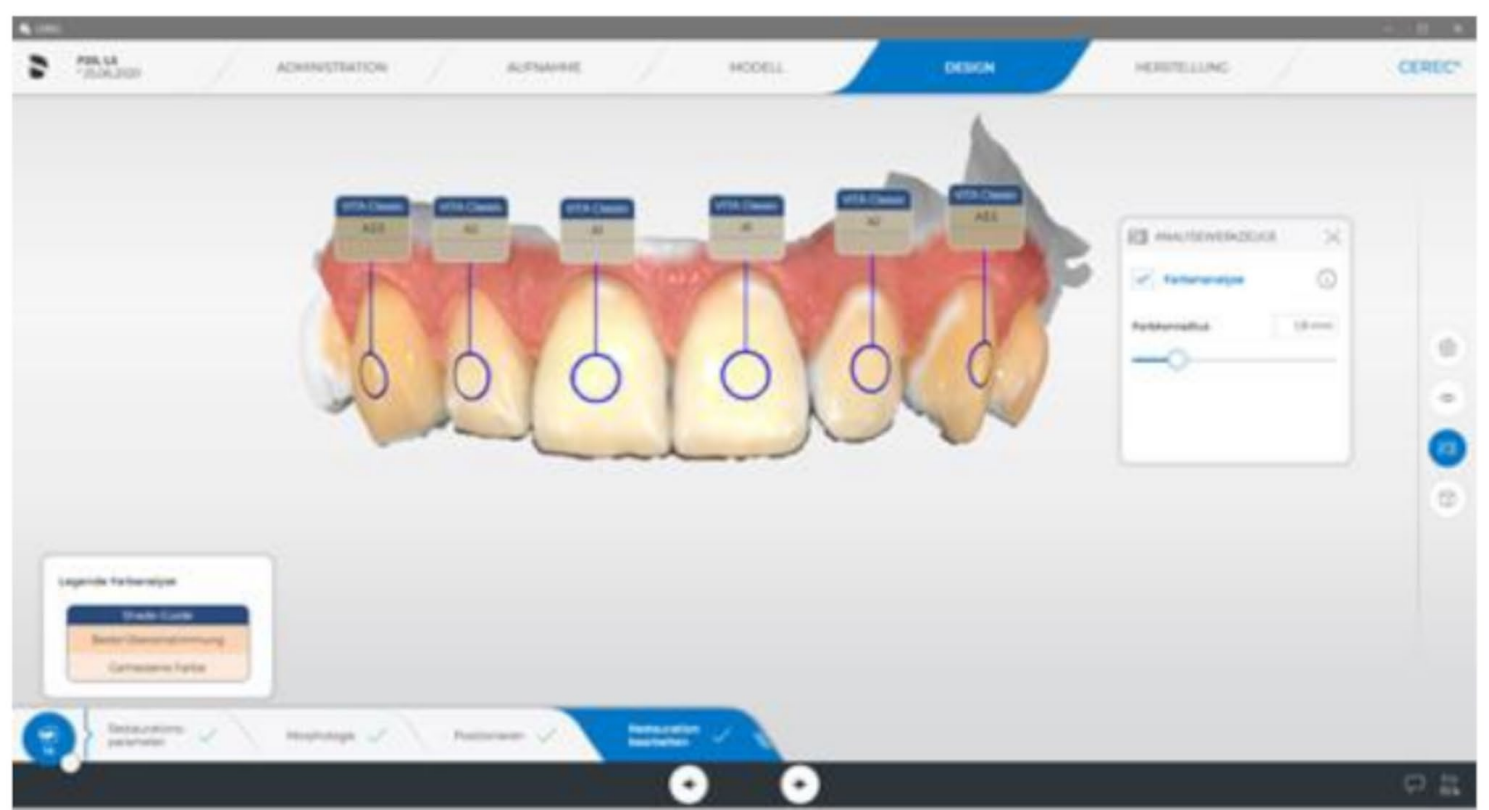
(S_2_) segment tooth shade measurement on the intraoral scan [13]

**Fig. 2:**
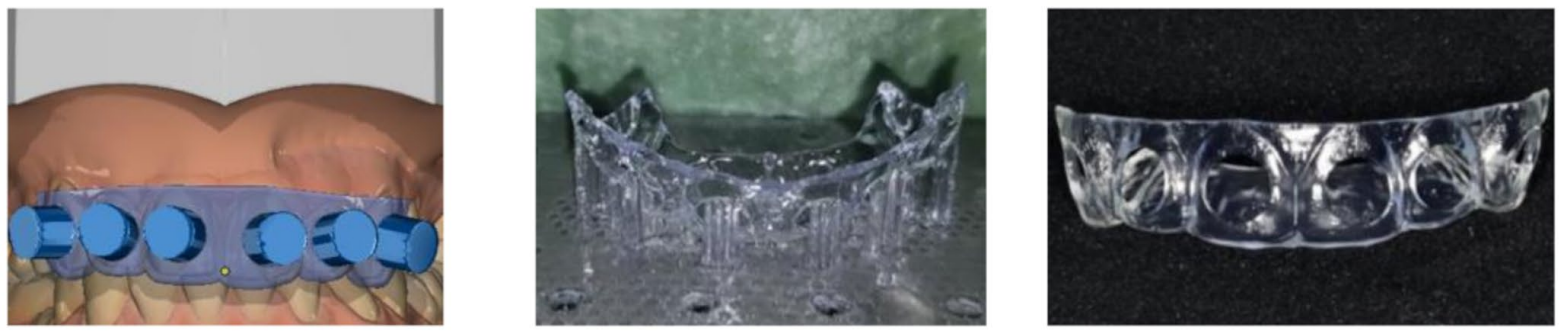
Splint construction and manufacturing [13]

**Fig. 3:**
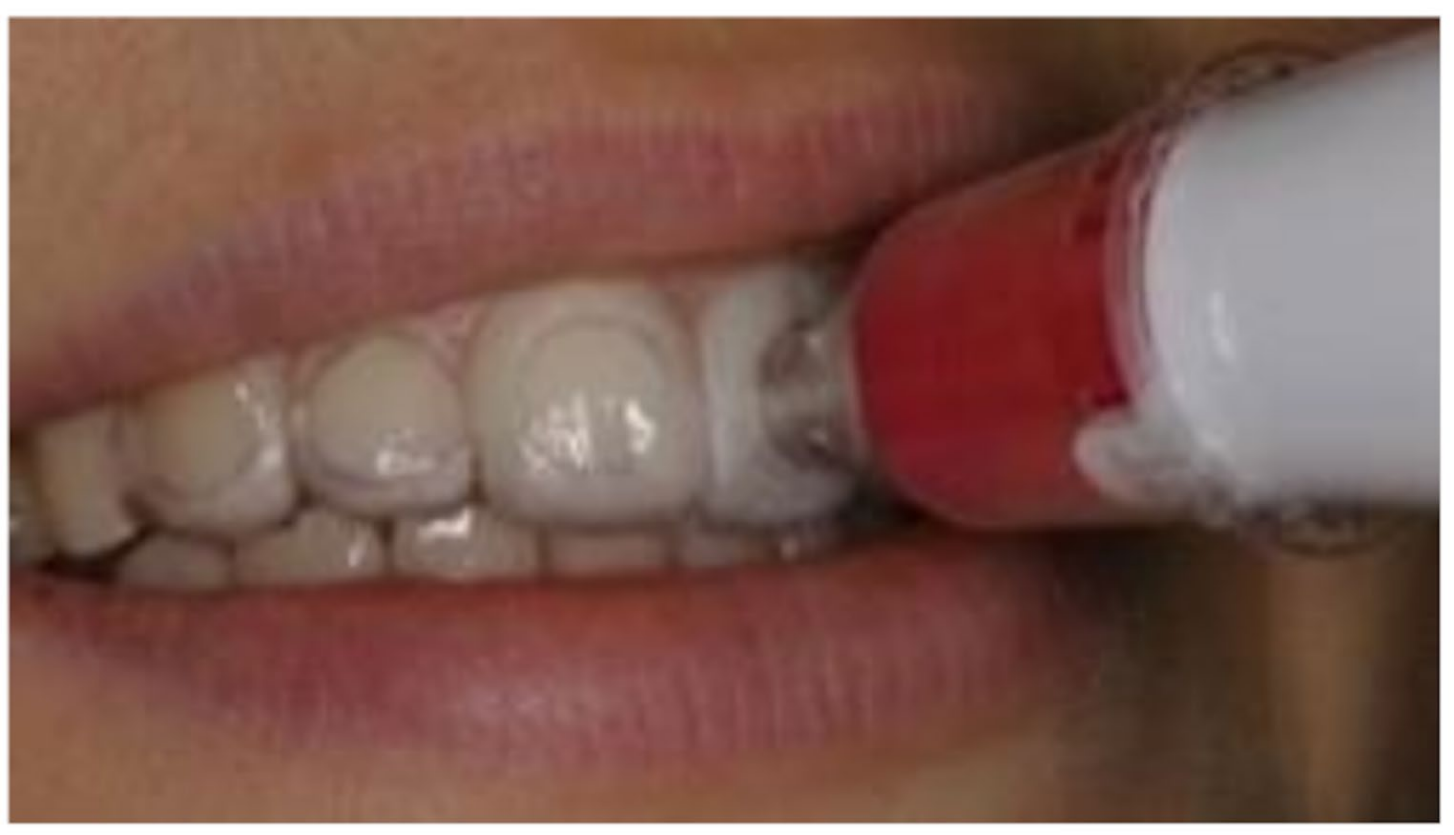
(S_2_) segment tooth shade measurement with the spectrophotometer [13]

The complete clinical procedure was performed under standardized conditions. The place of examination, the consistent methodical workflow, and factors such as constant lighting conditions were taken into account. The electronic tooth shade measurements were performed with the intraoral scanner CEREC Primescan AC and the spectrophotometer VITA Easyshade V. The Vita Easyshade V uses a contact measurement system with a 45°/0° geometry, where illumination occurs at a 45-degree angle and measurement is taken perpendicular (0°) to the surface [12]. The VITA Easyshade V calibrates by comparing light reflections from an in the base unit integrated ceramic calibration block against known reference values, performed before each measurement. Primescan uses an array of infrared LEDs for illumination while the scanning process and multiple cameras to capture the reflected light from various angles simultaneously. By scanning color fields in a calibration box and comparing the reflected values to known standards, calibration in our study was performed following the manufacture guidelines [13]. The tooth color was measured two times in the middle segment (S_2_) of the labial surfaces of tooth 13–23. The VITA classical A1-D4^®^ shade guide (VITA Zahnfabrik, Bad Säckingen, Germany) served as a reference system. The two-dimensional structure of this shade system enables the description of hue (category A to D) and lightness (group 1 to 4). After activating the color acquisition function of the intraoral scanner (Cerec Software 5.1.2, Dentsply Sirona), the scans were taken and the cursor was manually placed on the S_2_ segments of the teeth to be measured (Fig. 1). To ensure reproducibility of the measuring area (S_2_) in repeated spectrophotometric measurements, CAD / CAM-manufactured splints were constructed. In the “FA_Bonding 3D” and “Bonding Trays 3D” module of OnyxCeph³™ 3D Lab (Image Instruments GmbH, Chemnitz, Germany) these were planned with intraoral scans of the subjects (Fig. 2a-c). Using the 3D-DLP method (SHERAprint-ortho plus UV; SHERAeco-print 30, SHERA Werkstoff-Technologie GmbH & Co., Lemförde, Germany) these were printed for each participant (Fig. 3).

### Statistical methods

A variety of quality assurance and control methods recommended for use in the medical field were used [14]. Whereas reliability parameters, including the ICC, are relevant for group comparisons, e.g. comparing a bracketed group with a non-bracketed group (a single measurement per subject in each group), agreement parameters, including the standard error of measurement (SEM) and the smallest detectable color difference (SDCD = $\:1.96\text{*}\sqrt{2}\text{*}\text{S}\text{E}\text{M}\:\approx\:\:2.77\text{*}\text{S}\text{E}\text{M}$), are relevant for before-after comparisons, e.g. before and after brackets (at least two measurements per subject), which is an important conceptual difference [15]. Moreover, SEM and SDCD can be interpreted on the original scale d(0M1) based on units of ΔE, which is advantageous for clinical interpretation, especially for assessing clinical relevance.

For this purpose, all VITA classical color values were converted to the corresponding L*, C*_ab_, and h^0^ values of the CIE L*a*b* system using a conversion table according to Park et al. [16]. Park et al. [16] provide color conversation values under three standard illuminants (D_65_, A, and F2) on a reflection spectrophotometer (Color-Eye 7000 A; GretagMacbeth Instruments Corp). We used D_65,_ which represents a phase of daylight with a correlated color temperature of approximately 6500 K [16]. It’s the only conversion table available that provides a standardized method for converting VITA classical color values to the CIE L*a*b* system. To describe the distribution of repeated measurements, the mean and standard deviation were calculated as well as the pooled standard deviation. The agreement for ΔE < 2.7 /ΔE < 3.7 and d(0M1), was used to assess clinical relevance ($\:\varDelta\:\text{E}\:=\:\sqrt{\varDelta\:{L}^{2}\:+\:\varDelta\:{a}^{2}\:+\:\varDelta\:{b}^{2}}$) [10]. Bland-Altman plots [17, 18] - now considered the standard approach for assessing the agreement between two measurement methods widely used by researchers in various disciplines - were used for graphical evaluation [19]. Assuming independent teeth from the rater’s perspective, the classical plot shows the mean difference and the limits of agreement [17], here for d(0M1), which is the ΔE of each color shade from 0M1. The limits of agreement were also corrected for multiple teeth per subject [18]. Next to the limits of agreement (difference between measurements ± 1.96* standard deviation of the difference), the expected and observed number of observations outside these limits are given. In addition to systematic bias assessed by the global difference in Bland-Altman plots, we visualized local bias using local regression based on the Lowess smoother. To assess the reproducibility of two repeated measurements within the same tooth, the SEM and the SDCD were calculated [15]. This calculation of SDCD from SEM was restricted to an α level of 5% (corresponding to a factor of 1.96), although other α levels may be justified. For intra-method reliability, a guideline on the ICC leads to the two-way mixedeffects model with “absolute agreement” (as opposed to “consistency” [20]; not to be confused with “agreement” as opposed to “reliability” [15]), which yields the ICC_(2,1)_ according to the column for formulas for calculating the ICC in Table 3 of the guideline [20]. For inter-method reliability, a large discrepancy between ICC_(2,1)_ and the “consistency” ICC_(3,1)_ gives important information [45], which is supported by considering the ICC formulas [15, 20]. Since conventional ICCs assume independent teeth, a version of the ICC that corrects for multiple teeth per subject was computed from multilevel models [47]. The strength of ICC was interpreted using Byrt’s 1996 classification [14, 21]. Graphs and statistical analyses were performed using Stata software (release 17.0, Stata Corporation, College Station, TX, USA). To avoid overlapping of identical observations with the same coordinates within the Bland-Altman plots, all data points were jittered.

## Results

Of the initial 35 participants, all underwent spectrophotometric measurements, but due to random dropouts during the intraoral scanning process, only 22 intraoral scan measurements were ultimately evaluated.

### Shade characteristics

At first measurement, 8 different shades occurred for VITA Easyshade (A1: *n* = 71; A2: *n* = 27; A3: *n* = 43; A3,5: *n* = 23; A4: *n* = 1; B1: *n* = 12; B2: *n* = 25; B3: *n* = 6), at second measurement, 8 different shades occurred for VITA Easyshade (A1: *n* = 67; A2: *n* = 25; A3: *n* = 48; A3,5: *n* = 21; B1: *n* = 11; B2: *n* = 30; B3: *n* = 5; B4: *n* = 1). At first measurement, 7 different shades occurred for Primescan (A1: *n* = 56; A2: *n* = 20; A3: *n* = 16; A3,5: *n* = 2; B1: *n* = 19; B2: *n* = 10; B3: *n* = 7), at second measurement, 7 different shades occurred for Primescan (A1: *n* = 51; A2: *n* = 17; A3: *n* = 21; A3,5: *n* = 2; B1: *n* = 24; B2: *n* = 10; B3: *n* = 5).

### Intra-method agreement and reliability

**Fig. 4:**
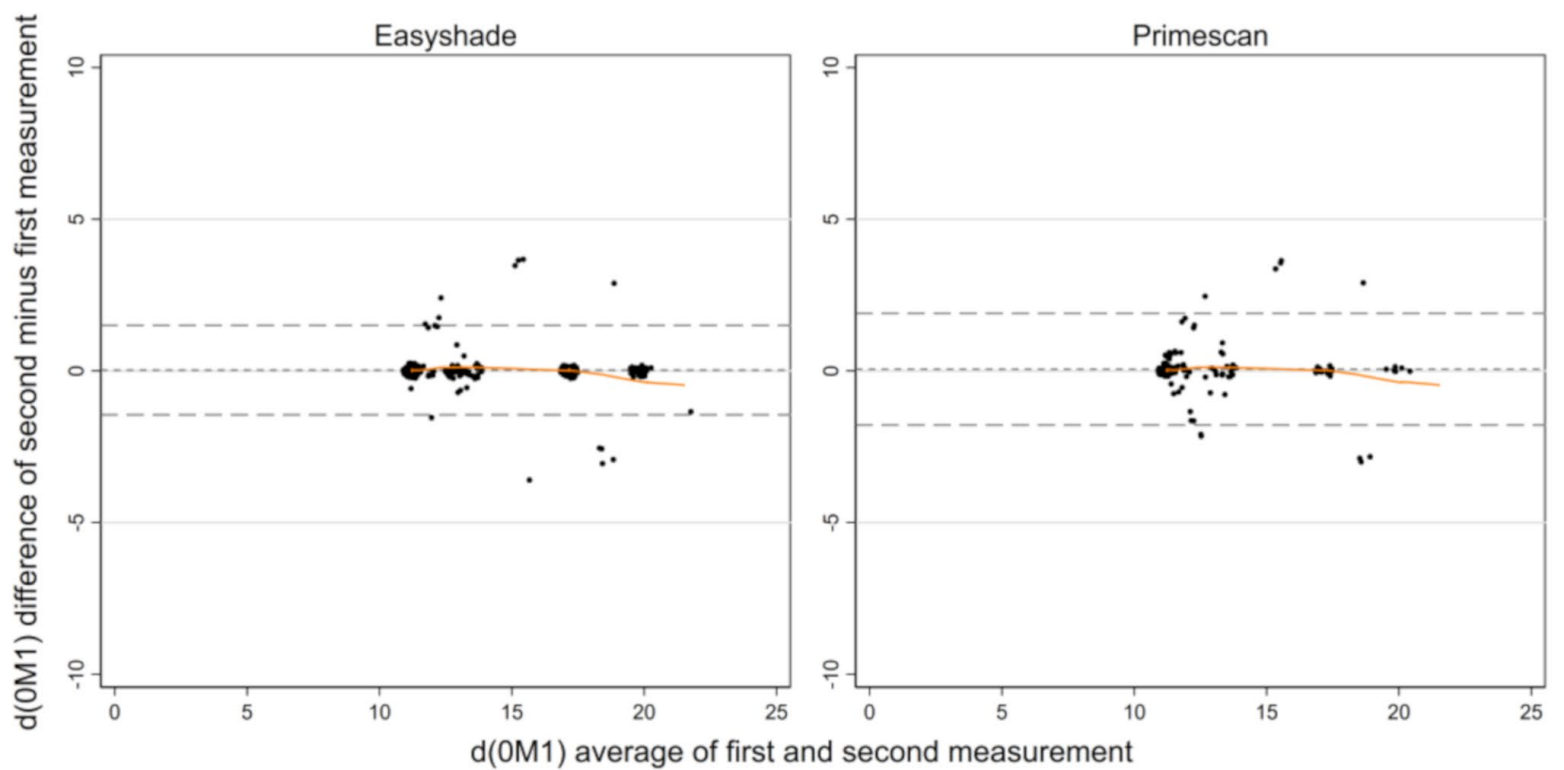
Bland-Altman plot with Lowess smoother (orange) to detect local bias for single tooth measurements of Easyshade and Primescan for d(0M1) for *n* = 208 and *n* = 130, respectively

The results already showed a very good agreement for ΔE < 2.7 for both methods (Table 1). For d(0M1), the spectrophotometric method showed a higher variability with a pooled standard deviation of 3.17 than the intraoral scanner with 2.67 (Table 2). Both methods already achieved an excellent agreement for d(0M1) < 2.7 (Table 2). In both Bland-Altman plots, the observed number of observations outside the limits of agreement was expectable; local bias was not detected by the Lowess smoother (Table 2; Fig. 4). The limits of agreement were not changed for the number of digits presented if adjusting for multiple teeth within subjects. For repeatability within the same tooth, the standard error of measurement of the intraoral scanner was larger than that of the spectrophotometric method; correspondingly, the SDCD was smaller for the spectrophotometric method (Table 2). For reliability of two different teeth, the ICC showed an excellent agreement for both methods (Table 2).

**Table 1:** Agreement and reliability of repeated measurements for two methods in terms of the distance from 0M1 related to a single tooth [13]

	Spectrophotometer/ Easyshade	Intraoralscan/ Primescan
	Value	Value
Number of paired observations	208	130
Mean distance (SD) d_1_ from 0M1 for the 1st measurement	14.2 (3.21)	13.1 (2.69)
Mean distance (SD) d_2_ from 0M1 for the 2nd measurement	14.3 (3.13)	13.2 (2.65)
Pooled SD of the 1st and 2nd measurement	3.17	2.67
Agreement within|d(0M1)| < 2.7, proportion (95% CI)	96.6 † (93.2–98.6)	94.6 † (89.2–97.8)
Agreement within|d(0M1)| < 3.7, proportion (95% CI)	100.0 † (98.2–100.0)	100.0 † (97.2–100.0)
Difference d_2_– d_1_ (standard deviation)	0.02 (0.75)	0.06 (0.94)
Limits of agreement	-1.45–1.50	-1.78–1.90
Number of observations outside the limits of agreement total (lower; higher); expected: 4–17 for *n* = 208 and 2–11 for *n* = 130	16 (6; 10)	10 (5; 5)
Largest mean d(0M1) value	21.5	20.1
Smallest mean d(0M1) value	11.2	11.2
Standard error of measurement _(2,1)_	0.531	0.663
Standard error of measurement _(3,1)_	0.531	0.663
SDCD_(2,1)_	1.47	1.84
ICC_(2,1)_ (95% CI)	0.97 † (0.96–0.98)	0.94 † (0.91–0.96)
ICC_tooth|subject_ (95% CI)	0.97 † (0.96–0.98)	0.94 † (0.91–0.96)

SD: standard deviation; CI: confidence interval; SDCD: smallest detectable color difference;

ICC: intraclass correlation coefficient

Classification for the interpretation of agreement

* fair (41–60); ** good (61–80); *** very good (81–92); † excellent (93–100)

Classification for the interpretation of the ICC:

* fair (0.41 − 0.60); ** good (0.61 − 0.80); *** very good (0.81 − 0.92); † excellent (0.93 − 1.00)

**Table 2:** Agreement of repeated measurements for two methods in terms of ΔE related to a single tooth [13]

	Spectrophotometer/ Easyshade	Intraoralscan/ Primescan
	Value	Value
Paired observations, number	208	130
Mean ΔE (standard deviation)	0.32 (0.94)	0.68 (1.19)
Agreement within ΔE < 2.7, proportion (95% CI)	92.8 ^***^ (88.4–95.9)	86.9 ^***^ (79.9–92.2)
Agreement within ΔE < 3.7, proportion (95% CI)	97.6 † (94.5–99.5)	97.7 † (93.4–99.5)

Classification for the interpretation of agreement:

*fair (41–60); ** good (61–80); *** very good (81–92); † excellent (93–100)

### Inter-method agreement and reliability

**Fig. 5:**
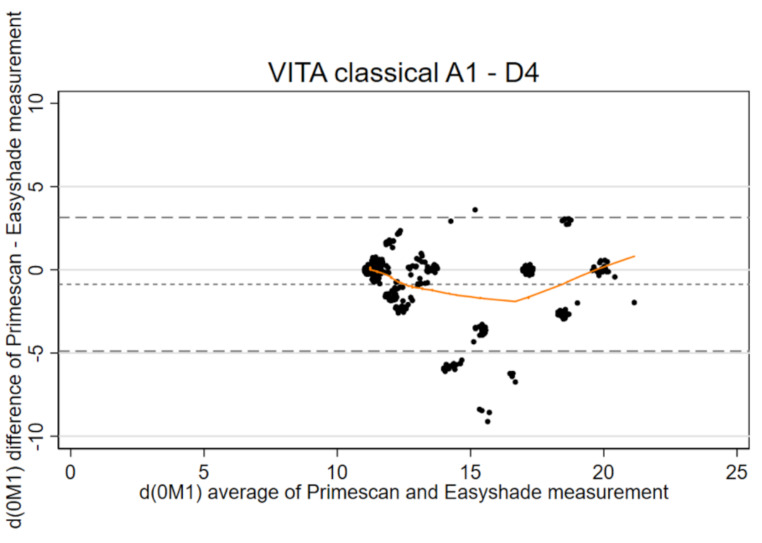
Bland-Altman plot with Lowess smoother (orange) to detect local bias for single tooth measurements of Easyshade vs. Primescan for d(0M1) for *n* = 338 [13]

The methods showed a good agreement for ΔE < 2.7 and a very good agreement for ΔE < 3.7 (Table 3). For d(0M1), the methods show a very good to excellent agreement (Table 3). According to the Bland-Altman plot, the methods differed systematically (Table 4; Fig. 5). Moreover, the observed number of observations outside the limits of agreement was slightly higher than expected (Table 4; Fig. 5). The limits of agreement were changed from − 4.88 to 3.15 (Table 4; Fig. 5) to -4.92–3.18 if adjusting for multiple teeth within subjects– without changing the observed number of observations outside the limits of agreement. The colors below the lower limit of agreement were A1, A2, and B1 (15 of 134, 5 of 48, and 5 of 55 measurements, respectively), which together were also prone to local bias detected by the Lowess smoother in the range of intermediate d(0M1) values, and the color above the upper limit of agreement was A3 (1 of 48 measurements). The ICC showed good agreement. There is no large discrepancy between ICC_(2,1)_ and ICC_(3,1)_, which means that the variance in the two methods is relatively small [15], supporting that intraoral scanners are an alternative to spectrophotometric methods.

**Table 3:** Comparing methods of measurements in terms of ΔE: Easyshade *versus* Primescan [13]

	within VITA classical A1-D4
	Value
Paired observations, number	338
Mean ΔE (standard deviation)	1.93 (2.19)
Agreement within ΔE < 2.7, proportion (95% CI)	66.3 ^**^ (61.0–71.3)
Agreement within ΔE < 3.7, proportion (95% CI)	83.4 ^***^ (79.0–87.2)

Classification for the interpretation of agreement:

* fair (41–60); ** good (61–80); *** very good (81–92); † excellent (93–100)

**Table 4:** Comparing methods of measurements of the distance from 0M1 related to a single tooth: Easyshade *versus* Primescan [13]

	within VITA classical A1-D4
	Value
Number of paired observations	338
Mean distance (SD) d_1_ from 0M1 for the Easyshade measurement	14.2 (3.15)
Mean distance (SD) d_2_ from 0M1 for the Primescan measurement	13.3 (2.78)
Agreement within|d(0M1)| < 2.7, proportion (95% CI)	85.5 ^***^ (81.3–89.1)
Agreement within|d(0M1)| < 3.7, proportion (95% CI)	92.3 ^***^ (88.9–94.9)
Difference d_2_– d_1_ (standard deviation)	-0.87 (2.05)
Limits of agreement	-4.92–3.18
Number of observations outside the limits of agreement total (lower; higher); expected: 9–25 for *n* = 338	26 (25; 1)
ICC_(2,1)_ (95% CI)	0.73 ^**^ (0.61–0.81)
ICC_(3,1)_ (95% CI)	0.76 ^**^ (0.71–0.80)
ICC_tooth|subject_ (95% CI)	0.72 ^**^ (0.65–0.78)

SD: standard deviation; CI: confidence interval; SDCD: smallest detectable color difference;

ICC: intraclass correlation coefficient

Classification for the interpretation of agreement

* fair (41–60); ** good (61–80); *** very good (81–92); † excellent (93–100)

Classification for the interpretation of the ICC:

* fair (0.41 − 0.60); ** good (0.61 − 0.80); *** very good (0.81 − 0.92); † excellent (0.93 − 1.00)

## Discussion

In the present study, the reproducibility of tooth shade determination on intraoral scans was determined in comparison with the spectrophotometric method. The reliability within both methods was very good, whereas the reliability across methods was too low for interchangeable use. Numerous studies have found that electronic methods are more accurate or reliable than visual measurements [22]. Especially spectrophotometers are proven to be reliable tooth shade measuring instruments [12, 23, 24] and several studies demonstrated their superiority [22, 25, 26]. In our study, a spectrophotometer was also used as a reference method compared to the intraoral scanning system.

Intra-method, Primescan, and VITA Easyshade achieved comparable results in terms of ΔE < 2.7/ ΔE < 3.7 with a very good/excellent agreement. Literature has already shown that the reproducibility of intraoral scanners is comparable to spectrophotometers [23, 27–29]. Mehl et al. [29] recorded no significant differences between an intraoral scanner (TRIOS Color) and four different spectrophotometers. The color measurements were carried out on 20 subjects on the central maxillary incisor and canine, each in the cervical, middle, and incisal third of the labial surface. All measurements were repeated three times and the CIE L*a*b* values were evaluated concerning ΔE. The results showed that the reproducibility of all methods (percentage agreements of 66.7% for TRIOS Color, 68.3% for VITA Easyshade Advance 4.0, 61.7% for SpectroShade and 71.7% for SpectroShade Micro) were comparable. Brandt et al. [23] compared an intraoral scanner (TRIOS Color) with a spectrophotometer (VITA Easyshade Advance 4.0). The tooth color in the middle segment was determined three times on 20 central maxillary anterior teeth of patients between the ages of 20 and 53 years. After converting all measured colors into L*a*b* and L*C*h* values, the evaluation was carried out with regard to ΔE < 6.8, and the intra-individual reliability was determined using the ICC. Both the intraoral scanner and the spectrophotometric method achieved good to very good intra-individual agreement within repeated measurements. Due to the reference to d(0M1) chosen in our study and a perceptibility threshold of ΔE < 2.7/ ΔE < 3.7 with a higher sensitivity compared to ΔE < 6.8, there is only a limited comparability of the results. Nevertheless, the study confirms our results. Rutkunas et al. [28] examined the in vivo tooth color determination with an intraoral scanning system in the middle third of 120 maxillary anterior teeth on 20 patients aged 20 to 23 years. A spectrophotometer (SpectroShade) was used as a reference instrument. To determine the reproducibility each tooth was repeatedly measured five times and ΔE < 3.7 was calculated. The results showed that the reproducibility of the intraoral scanner (90.33% for VITA 3D-MASTER and 87.17% for VITA classical) is comparable to the spectrophotometer (92% for VITA 3D-MASTER and 93.5% VITA classical).

Both methods represent a good agreement concerning ΔE < 2.7 and a very good agreement for ΔE < 3.7. The results also show a very good agreement for d(0M1) < 2.7 and a very good to excellent agreement for d(0M1) < 3.7. Regarding d(0M1), the results for the inter-method reproducibility show a good agreement (ICC_(2,1)_ 0.73; ICC_(3,1)_ 0.76). In other studies, neither the interindividual comparison nor d(0M1) as a reference was used, so the comparison of the results in the literature is only possible to a limited extent [10]. Ebeid et al. [30] examined the accuracy and reproducibility of three different intraoral scanning systems (TRIOS; Omnicam and Primescan) compared to a spectrophotometer (VITA Easyshade V). The reproducibility of the color measuring instruments after repeated measurements was overall less than 55%. There were significant differences in the accuracy of the individual electronic color determination results (78% for the spectrophotometer, 66% for the TRIOS, 63% for the Primescan, and 57% for the Omnicam). The authors also recommend the visual method when using electronic color-determination instruments. By comparing three different intraoral scanners a broad perspective on device performance for comparison which was not appropriate was offered.

Our study assessed the inter-method reproducibility and found good to very good agreement. Due to the different statistical methods used to evaluate reproducibility, the results are only partially comparable. Statistics for repeated tooth shade measurements of intraoral scanning systems differ. The percentage of agreement [29, 30], the median value [28], or ΔE values [23, 28, 29] are used for reproducibility. In our study, a perceptibility threshold of ΔE < 2.7 and ΔE < 3.7 was used. Under experimental conditions, ΔE = 1 is referred as the limit, to which the human eye can perceive [24, 31, 32]. For the registration of color differences under clinical conditions, ΔE must assume a higher value. Therefore threshold values of ΔE = 2.7 [33], ΔE = 3.3 [34], or ΔE = 3.7 [35] are described in the literature. A systematic review concluded that acceptance thresholds of ΔE = 2.0 to ΔE = 4.0 are mostly used [31].

In our study, the standard error of measurement was smaller for the spectrophotometric method compared to the intraoral scanning system. Random and systematic errors may have an impact on instrument reproducibility. Furthermore, we calculated with d(0M1) in addition to ΔE [10]. Thus, the SDCD, Bland-Altman plots, and reliability statistics, including versions of the ICC, could also be evaluated [3]. A possible reason for the superiority of the spectrophotometric method could be that there is no standardized scanning method for detecting tooth shades with intraoral scanning systems. The scanning angle and the distance of the camera to the tooth surface are hardly controllable [36]. The study design can be considered as a factor that influences the results. In various studies, the tooth color determination function of intraoral scanning systems was evaluated in vitro [24, 30] and in vivo [23, 28, 29]. In vivo, studies with tooth color measuring instruments are more complicated than studies under standardized laboratory conditions because of the numerous influencing factors of the intraoral environment [26]. Dozic et al. [26] investigated the influence of in vitro and in vivo environments and concluded that higher reliability occurred under in vitro conditions. Factors such as ambient lighting do not influence spectrophotometric measurements [37]. For intraoral scanning systems, there is no clear consensus. Reyes et al. [38] concluded that ambient lighting does not influence intraoral scanners. Revilla-Leon et al. [39] found a significant influence of lighting conditions on the color determination function of an intraoral scanner. In addition to the different device functional principles, it must be taken into account that the intraoral scanning system records the entire tooth surface including the cervical and incisal area. For example, gingival reflections can be mentioned as a possible influence on color measurement using an intraoral scanning system. In contrast, the VITA Easyshade is a point-measuring device, so these factors have less influence.

Sirintawat et al. [24] investigated the reproducibility of the TRIOS 3 intraoral scanner compared to the VITA Easyshade Advance V. The color of 30 ceramic crowns was measured, the L*a*b* values were determined, and ΔE < 6.8 was used as the clinical acceptance threshold. The results showed excellent values for the intraoral scanning system (ICC of 0.996 for L*, 0.994 for a* and 0.884 for b*) and the spectrophotometric method (ICC of 0.989 for L*, 0.987 for a* and 0.999 for b*). Brandt et al. [23] compared the TRIOS Color scanner with the VITA Easyshade Advance 4.0. Tooth shades were determined on 20 central maxillary anterior teeth of patients, converted into L*a*b* and L*C*h* values, and evaluated concerning ΔE < 6.8. Both methods achieved good to very good intra-method agreement. Due to the different perceptibility thresholds of ΔE < 2.7/ ΔE < 3.7 with a higher sensitivity compared to ΔE < 6.8, the comparability is limited. Moreover, all measurements were taken on six maxillary anterior teeth to reduce irregularities of the measurement surface [16, 40]. As described by Karamouzos et al. [40] in addition to the central maxillary incisors, upper lateral incisors, and upper canines show significantly smaller color differences (∆E values). In our study measurements were taken in the middle segment of each tooth (S_2_). In the incisal segment, the translucent enamel, and in the cervical part, the gingiva influences the measurement [41] and the central area of the crown represents the tooth color best [42, 43]. Karamouzos et al. [40] showed that the central area of the middle segment provides the most accurate results. Furthermore, the measurement position can be challenging due to the uneven color properties of the teeth, which is associated with the complex layering of the enamel and dentin [44]. According to the reproducibility, the measurement position during repeated measurements is relevant. Positioning splints can be used to control potential positioning errors during repeated measurements [44]. Yilmaz et al. [27] also achieved reproducibility in their study by using positioning splints made of acryl. In our study, the VITA Easyshade was positioned for all participants with individually designed and 3D-printed splints during repeated measurements to achieve reproducible measurement positions.

Our in vivo study has several limitations such as the sample size discrepancy: There was a dropout of 13 participants for intraoral scans, resulting in only 22 intraoral scan measurements compared to 35 spectrophotometric measurements. Furthermore, the in-vitro design does not capture the complexities of intraoral color determination in clinical situations. Our study, conducted in vivo, delivers results more reflective of clinical dental reality. Nevertheless, our study must account for variables related to patients, such as movement or saliva, which could influence the accuracy of the measurements. While our study is more closely aligned with clinical practice, the approach taken by Ebeid et al. [30] offers a more controlled environment for comparison. Both methods provide valuable insights, and future research could benefit from integrating the strengths of both designs. Furthermore, the age distribution of the participants must be considered, because an average age of 27 years is not representative of the population as a whole, as the tooth color becomes darker and yellower with increasing age [42]. Our color measurements using the VITA classical shade system did not encompass the full range of possible values, resulting in a notable underrepresentation of C and D shades among our subjects. The color space was limited because of the selected participants and the generalizability is limited because of the limited color space observed. As reference systems, the most widely served system VITA classical was used in our study [9, 10, 24]. While we did not incorporate the VITA 3D Master system due to our focus on the more commonly used 2D system, we acknowledge its potential. This corresponds to an opportunity for further investigation to provide valuable insights. In general, the software of intraoral scanners does not provide direct L*a*b* values but rather the VITA classical values. These L*a*b* values must be calculated from the color scale values using conversion tables that vary from study to study [24]. In our study, all VITA classical color values were converted to the corresponding L* and C*ab values of the CIE L*a*b* system using a conversion table according to Park et al. [16]. The geometry of the spectrophotometer used by Park et al. [16], which includes an integrating sphere for diffuse illumination, is different. These different geometric configurations have a direct influence on the comparability and is a limitation for the use of this conversion table. Nevertheless, this table is the only conversion table available for a standardized translating, making it a valuable tool for studies requiring such conversions. A further limitation might be that we used multiple teeth of the same subjects. However, there were no relevant differences between unadjusted and subject-adjusted limits of agreement [18]. Moreover, (in)dependence is an assumption and depends on the purpose rather than it is a fact; the purpose here is rather a statement about the method than the teeth. In other words: whereas it is justified to assume dependent observations for a human rater, it is not so for a machine. Studies that address any of these outlined limitations in their research design would be of particular scientific interest.

Notwithstanding these limitations, the study has some methodological strengths. Rarely used in dentistry are advanced approaches to reliability (ICC) and agreement (Bland-Altman approach), which were developed decades after the corresponding basic approaches [45, 46], namely approaches to adjust for multiple observations within subjects [18, 47], as done here. Another strength is that we used a variety of appropriate methods with different purposes and different assumptions to meet the intent of the 2016 American Statistical Association statement [48, 49], which emphasizes the importance of checking assumptions and sensitivity analyses for the interpretation of results [50], thereby excluding the often-used Pearson’s correlation coefficient [14]. The methods used here complement each other to provide a more complete picture of aspects of measurement error (reliability and agreement, dimensionless and derived from units of the original measurement scale, standard and advanced approaches, statistical and graphical methods, systematic and local bias).

## Conclusion

Within the limitations of the study both methods achieve comparable intra-method reproducibility concerning ΔE and d(0M1). Inter-method the VITA Easyshade achieved better results. Intraoral scanning systems represent a reproducible method for clinical tooth shade determination.

## Data Availability

No datasets were generated or analysed during the current study.
